# Twitter Social Media is an Effective Tool for Breast Cancer Patient Education and Support: Patient-Reported Outcomes by Survey

**DOI:** 10.2196/jmir.4721

**Published:** 2015-07-30

**Authors:** Deanna J Attai, Michael S Cowher, Mohammed Al-Hamadani, Jody M Schoger, Alicia C Staley, Jeffrey Landercasper

**Affiliations:** ^1^ UCLA Health Burbank Breast Care Burbank, CA United States; ^2^ Allegheny Health Network Pittsburgh, PA United States; ^3^ Gundersen Health System Department of Medical Research La Crosse, WI United States; ^4^ Women With Cancer The Woodlands, TX United States; ^5^ Akari Healthcare Charlestown, MA United States; ^6^ Gundersen Health System Norma J. Vinger Center for Breast Care La Crosse, WI United States

**Keywords:** breast cancer, education, social support, social media, patient outcome assessment

## Abstract

**Background:**

Despite reported benefits, many women do not attend breast cancer support groups. Abundant online resources for support exist, but information regarding the effectiveness of participation is lacking. We report the results of a Twitter breast cancer support community participant survey.

**Objective:**

The aim was to determine the effectiveness of social media as a tool for breast cancer patient education and decreasing anxiety.

**Methods:**

The Breast Cancer Social Media Twitter support community (#BCSM) began in July 2011. Institutional review board approval with a waiver of informed consent was obtained for a deidentified survey that was posted for 2 weeks on Twitter and on the #BCSM blog and Facebook page.

**Results:**

There were 206 respondents to the survey. In all, 92.7% (191/206) were female. Respondents reported increased knowledge about breast cancer in the following domains: overall knowledge (80.9%, 153/189), survivorship (85.7%, 162/189), metastatic breast cancer (79.4%, 150/189), cancer types and biology (70.9%, 134/189), clinical trials and research (66.1%, 125/189), treatment options (55.6%, 105/189), breast imaging (56.6%, 107/189), genetic testing and risk assessment (53.9%, 102/189), and radiotherapy (43.4%, 82/189). Participation led 31.2% (59/189) to seek a second opinion or bring additional information to the attention of their treatment team and 71.9% (136/189) reported plans to increase their outreach and advocacy efforts as a result of participation. Levels of reported anxiety before and after participation were analyzed: 29 of 43 (67%) patients who initially reported “high or extreme” anxiety reported “low or no” anxiety after participation (*P*<.001). Also, no patients initially reporting low or no anxiety before participation reported an increase to high or extreme anxiety after participation.

**Conclusions:**

This study demonstrates that breast cancer patients’ perceived knowledge increases and their anxiety decreases by participation in a Twitter social media support group.

## Introduction

Reporting that United States cancer care was “in crisis,” the Institute of Medicine highlighted several critical areas for improvement in 2013 including the development of better methods for patient education, communication, shared decision making, and support [1]. They recommended 10 distinct goals, 3 of which are relevant to social media: providing patients with more “understandable information,” psychosocial support, and to collect more patient-reported outcomes [1].

Traditional on-campus patient support groups are valuable and have been recommended by the National Accreditation Program for Breast Centers and other organizations in the United States [2,3]. However, traditional support groups may not be available for all patients. Patients may not participate because of inconvenient meeting times, transportation and child care issues, perception that their needs are not being specifically addressed, or reluctance to share their feelings or stories in public [4,5]. As an alternative, many women utilize various Internet and social media resources for medical information, advice, and support, such as blogs, chat groups, Facebook, and Twitter [4-7]. Social media is inherently bidirectional, interactive, and patient-driven in contrast with older models of health care education and decision making that are unidirectional and paternalistic.

Twitter is an online social networking service created in 2006 that enables users to send and read 140-character messages or “tweets.” As of December 2014, Twitter had more than 280 million monthly active users [8]. Twitter has increasingly been embraced by patients as a way to share information and connect with other patients with similar concerns and conditions [7,9,10]. Disease-specific communities and chats have developed around “hashtags” (the # symbol followed by a word or acronym). Twitter chats now exist for patients with breast, lung, gynecologic, and pancreatic cancers, as well as multiple myeloma [11,12].

The Breast Cancer Social Media tweet chat (#BCSM) was initiated July 4, 2011, by 2 breast cancer survivors (authors JMS and ACS). Another author (DJA) became a comoderator in October 2011. The goal was to provide credible, evidence-based information and support for anyone affected by breast cancer. The chats occur on a weekly basis and cover all aspects of breast cancer screening, diagnosis, treatment, and survivorship. Specific medical advice is not provided and self-promotion or negativity toward any participant is actively discouraged.

Because of the growth and popularity of the #BCSM community, we sought to investigate the efficacy of Twitter social media to provide education and support to breast cancer patients. The study described in this paper is a pilot investigation assessing whether Twitter social media can provide education and psychosocial support to breast cancer patients.

## Methods

A participant survey was developed to determine tweet chat participant characteristics and to assess the effectiveness of #BCSM for education and anxiety. Ten patient characteristics and 15 patient outcome domains were included. A 5-point Likert-type bipolar-scaled response was provided to survey participants for questions regarding whether their participation in #BCSM resulted in increased understanding for different domains of care and treatment. In addition, participants were queried for level of anxiety pre- and post-Twitter engagement, safety and comfort of participation in #BCSM, and motivation toward future advocacy and volunteer activities.

Surrogate measures of the impact of #BCSM in the Twitter community were obtained to include the number of tweets, the number of followers, and the product of multiplying these numbers, a measurement of the potential reach of #BCSM tweets [13].

A waiver of informed consent was obtained from the Gundersen Health System Institutional Review Board for the deidentified survey that was offered to Twitter participants. The survey link (Survey Monkey) was posted from April 14 to 24, 2014, on Twitter, the #BCSM blog, and the #BCSM Facebook page [14,15]. After survey closure, statistical analysis was performed to search for associations between patient characteristics and educational improvement for multiple domains of care. The majority of respondents were found to be in a single demographic group and some responders did not answer every survey question. This caused the sample sizes between different patient characteristics to be unbalanced, limiting statistical comparisons because of bias issues. Therefore, we limited analytic reporting to frequencies and percentages except for respondent-reported extreme or high anxiety before and after participation in the #BCSM tweet chats. The McNemar test was used for this comparison. Lastly, the data on overall Twitter participation with #BCSM and its impact based on number of impressions were determined.

## Results

There were 206 survey responders. Respondent demographic and background information are presented in Table 1.

**Table 1:** Twitter participant characteristics (N=206).

Characteristic		n (%)^a^
**Age (years)**		
	≤24	1 (0.5)
	25-34	21 (10.2)
	35-44	46 (22.3)
	45-54	69 (33.5)
	55-64	57 (27.6)
	65-74	11 (5.3)
	≥75	1 (0.5)
**Sex**		
	Male	15 (7.2)
	Female	191 (92.7)
**Community population size**		
	<10,000	15 (7.3)
	10,000-100,000	46 (22.3)
	100,001-1,000,000	72 (34.9)
	>1,000,000	73 (35.4)
**Race/ethnicity**		
	White (includes Latino and Hispanic)	189 (91.7)
	Black or African American	4 (1.9)
	North, South, or Central American, Native Indian, or Alaskan native	1 (0.5)
	Native Hawaiian or other Pacific Islander	2 (1.0)
	Asian	5 (2.4)
	Other	5 (2.4)
**Highest level of education**		
	Primary school	0 (0.0)
	Some high school, but no diploma	0 (0.0)
	High school diploma (or GED)	1 (0.5)
	Some college, but no degree	22 (10.7)
	2-year college degree	11 (5.3)
	4-year college degree	59 (28.6)
	Graduate-level degree	111 (53.9)
	None of the above	2 (0.9)
**Previous or current treatment for breast cancer**		
	Yes	143 (69.4)
	No	63 (30.5)
**If not been treated for breast cancer, which category describes you**		
	Caregiver, family, friend, or spouse to breast cancer patient	17 (25.4)
	Clinical health professional or researcher	25 (37.3)
	Other	25 (37.3)
**Length of participation in chats (months)**		
	<6	59 (28.6)
	6-12	54 (26.2)
	>12	93 (45.1)
**Engagement with #BCSM community outside of Twitter**		
	Yes	141 (77.0)
	No	42 (22.9)
**Participation in in-person support groups**		
	Yes	79 (43.2)
	**No**	**104 (56.8)**

^a^ Not all response categories add up to 206 total survey responders because not all participants answered every question.

A total of 92.7% (191/206) of respondents were female and 69.4% (143/206) of respondents were breast cancer patients. Other respondents included family, friends, advocates, surgeons, medical oncologists, radiation oncologists, clinical psychiatrists, genetic counselors, and physical therapists. Respondent-reported changes in level of understanding and education in multiple domains of care after tweet chat participation were assessed. Survey participants were asked whether participation “provided a safe and welcoming forum for support and education.” Of 183 respondents to this question, 116 (63.4%) “strongly agreed,” 44 (24.0%) “somewhat agreed,” 12 (6.5%) were “neutral,” 8 (4.4%) “somewhat disagreed,” and 3 (1.6%) “strongly disagreed.” As a result of #BCSM, 52 of 183 responders (28.4%) reported subsequent volunteer efforts. For analysis of respondent reports of anxiety, survey responders reporting on less than 50% of survey questions were excluded. The 189 remaining responders were analyzed for their recall levels of reported high/extreme anxiety before and after participation in the #BCSM tweet chats. We found a significant decrease in the proportion of respondents with extreme/high anxiety level from 43 of 153 patients (28.1%) to 14 of 152 (9.2%, *P*<.001). Also, no respondents who initially reported “low or no” anxiety before participation reported an increase to “high or extreme anxiety” after participation. See Table 2 for other respondent answers.

Overall Twitter participation with #BCSM and its potential reach based on number of impressions is shown in Table 3 [13].

**Table 2:** Improvement in knowledge level after Twitter participation (N=206).

Knowledge domain	Response, n (%)^a^				
	Definitely yes	Somewhat yes	Not sure	Somewhat no	Definitely no
Breast cancer type^b^	66 (34.9)	68 (35.9)	22 (11.6)	16 (8.4)	17 (8.9)
Surgery and reconstruction	38 (20.1)	67 (35.4)	33 (17.5)	23 (12.2)	28 (14.8)
Radiation treatment	31 (16.4)	51 (26.9)	47 (24.9)	25 (13.2)	35 (18.5)
Breast imaging	39 (20.6)	68 (35.9)	33 (17.5)	21 (11.1)	28 (14.8)
Metastatic stage 4 cancer	95 (50.2)	55 (29.1)	17 (8.9)	9 (4.7)	13 (6.9)
Clinical trials	45 (24.3)	80 (42.3)	30 (15.9)	17 (8.9)	17 (8.9)
Genetic risk	36 (19.0)	66 (34.9)	45 (23.8)	24 (12.6)	18 (9.5)
Survivorship^c^	99 (52.4)	63 (33.3)	11 (5.8)	8 (4.2)	8 (4.2)
Advocacy and fundraising	70 (37.0)	66 (34.9)	33 (17.5)	13 (6.9)	7 (3.7)
Healthy lifestyle^d^	40 (21.2)	64 (33.9)	44 (23.3)	18 (9.5)	23 (12.2)
My impact on others	76 (40.2)	70 (37.0)	25 (13.2)	9 (4.8)	9 (4.8)
Seek second opinion^e^	29 (15.3)	30 (15.9)	19 (10.1)	18 (9.5)	49 (25.9)
Overall education with “your” cancer^e^	31 (16.4)	84 (44.4)	42 (22.0)	0 (0.0)	0 (0.0)
Overall education with any breast cancer	58 (30.7)	95 (50.2)	35 (18.5)	1 (0.5)	0 (0.0)

^a^ The number of respondents included in the denominator differs for each survey question because some respondents did not answer every question.

^b^ Understanding of estrogen receptors (ER) and progesterone receptors (PR), HER2, triple-negative types and meaning.

^c^ Understanding posttreatment follow-up side effects, lymphedema (arm swelling), cognitive impairment from chemotherapy, sexuality, grief, death, or other.

^d^ Diet, exercise, lifestyle habits.

^e^ Does not equal to 100% because the question was not applicable to the noncancer participants.

**Table 3:** Annual trends of Twitter participation with #BCSM.

Twitter participation	Year			
	2011	2012	2013	2014
Impressions,^a^ n	101,263,199	309,657,740	295,718,132	343,586,925
Impressions per user, mean	48,359	71,963	35,853	24,220
Tweets,^b^ n	28,275	69,505	84,614	85,972
Tweets per user, mean	13.5	16.2	10.3	6.1
Users,^c^ n	2094	4303	8248	14,186

^a^ The (number of tweets)*(number of followers), a measurement of the potential reach of a tweet.

^b^ Tweet that went out tagged with #BCSM.

^c^ Unique individuals who posted anything with #BCSM.

## Discussion

Major health care policy stakeholders in the United States endorse a model of patient care that is not solely provider- or institution-directed [1,16-22]. These stakeholders recognize that differences may exist between providers, payers, and patients regarding what constitutes good care and how to measure it [23]. Consequently, they have developed tools and resources that promote and measure patient-centered care, such as the Patient Center for Outcomes Research, the Consumer Assessment of Healthcare Providers and Systems (CAHPS) surveys, and breast surgery patient surveys [18-21]. Unfortunately, the existing resources for measuring patient quality of life, such as CAHPS surveys, have limitations because they require significant infrastructure and financial support for their use. In contrast, social media has the ability to aid patient centeredness, report patient outcomes, and provide breast specialists with patient perceptions of gap in care with limited financial and information technology investment. Social media is user-friendly and popular for health care consumers as evidenced by its rapid growth. Moreover, because of the widespread use and potential inclusiveness of nearly all patient demographic groups in social media, it is fertile territory for future investigations regarding identification of gaps in care, along with its potential to measure the success of patient education and support. This study is an initial investigation into the ability of Twitter social media to improve breast cancer patient education and support. Our primary aim was to determine patient’s perception of level of benefit of participation.

Only a few other reports of benefits of participation in an online social media-based support group have been published, although cancer survivors report the Internet as the second most important source for cancer information after their health care professional [6,24]. Online support groups have been shown to fill gaps in supportive care by meeting needs of some breast cancer survivors [25].

Approximately 20% to 30% of breast cancer survivors demonstrate measurable signs of anxiety and/or depression in the year following diagnosis [26,27]. Although the symptoms are most pronounced during the first year after diagnosis, up to 15% have depressive symptoms 5 years after diagnosis. Our study demonstrated an association between a reduction in breast cancer-perceived anxiety and participation in the #BCSM tweet chats. Other measures of success were also demonstrated. Twitter participants reported improved knowledge about multiple domains of care through the continuum of the cancer care timeline, inferring traditional educational resources may not have been sufficient. Despite this success, Twitter and other forms of social media cannot replace traditional office- and hospital-based resources for education and emotional support. In our survey, nearly 1 in 5 Twitter participants reported no improvement in education and 9% had persistent high anxiety despite #BCSM suggesting that Twitter participation was not sufficient to address education and anxiety needs in these patients. Therefore, the role of social media is to compliment but not replace current practice. It is reassuring that nearly 90% of Twitter followers reported the #BCSM chats as a “safe and comfortable” environment, given that more than half of participants reported no involvement in any other support group. A novel finding of this study is the observation that many responders to our survey were motivated by #BCSM to participate in advocacy or volunteer efforts.

There are many limitations to this pilot social media study. Our patient survey has not undergone formal reliability and validity testing and the survey format is subject to recall bias. We cannot determine if recall bias occurred because our survey was open for participation at only one snapshot in time. In addition, there was no control group of patients, with similar demographic characteristics, who had never participated in Twitter and were concurrently surveyed for their level of education and anxiety over a similar time period. Our survey cohort was homogenous, mostly white, and well educated. This lack of diversity prevents any statistical comparisons between demographic groups for Twitter’s ability to educate or ameliorate anxiety. The lack of diversity also does not allow extrapolation of study results to other patient populations to include non-Twitter users who may have differences in their demographic profile and cancer status compared to Twitter users.

It is unclear if the respondents to the survey were truly representative of the #BCSM community at large. Given the public nature of Twitter, there is no way to fully assess the participant composition of any Twitter chat. Of the survey respondents, 45% reported that they observe the chats but do not participate. “Lurking” (observing but not actively participating) on social media forums is a well-described phenomenon, although it has been reported that lurkers obtain the same benefits in terms of empowerment as those who actively participate [28,29].

Despite these limitations, our results represent an important first step toward development of more online patient education and support communities. An association between Twitter participation and improvements in patient self-reported knowledge and anxiety was identified. Compared to other cancer patients, those with breast cancer are more likely to seek online information about their condition [30]. Health care providers utilizing social media serve as patient advocates, assisting patient and family understanding of their disease and its treatment. Physician participation in online patient communities can help counter inaccurate and sometimes dangerous information. Other benefits of physician use of social media have been described [31].

Since the first #BCSM tweet chat on July 4, 2011, more than 160 chat hours have been logged. Chat activity, measured by the overall number of tweets, has increased each year as has the unique number of participants. Other cancer-related tweet chats have developed (Table 4), including sites for lung cancer, gynecologic cancers, multiple myeloma, and pancreatic cancer.

**Table 4:** Cancer-related tweet chat sites.

Cancer type	Hashtag (#)	Physician moderator(s)	Twitter handle
Breast	#BCSM	Dr Deanna J Attai	@DrAttai
Lung	#LCSM	Dr H Jack West	@JackWestMD
		Dr. David T. Cook	@UCD_ChestHealth
Gynecologic	#GYNCSM	Dr Don Dizon	@drdonsdizon
		Dr Merry Markham	@DrMarkham
		Dr Rick Boulay	@journeycancer
Multiple myeloma	#MMSM	Dr Mike Thompson	@mtmdphd
Pancreatic	#PANCSM	Dr Niraj Gusani	@NirajGusani
		Dr Mark Bloomston	@pancdoc
		Dr Matthew HG Katz	@mkatzmd
		Dr Diane Reidy-Lagunes	@DianeReidyLagun

A unique aspect of these chats is patient-physician involvement; in fact, most are comoderated by patients and physicians. The patients participating are seeking credible evidence-based information—something that may be lacking in other online forums. For physicians wary of the social media environment, #BCSM and the other cancer-related tweet chats provide an example of positive patient-physician engagement.

By surveying Twitter participants, we demonstrated proof of concept of improved patient knowledge regarding their disease-specific condition and management. Further investigations are warranted to explore its capability to provide increased patient knowledge, psychosocial support, and meaningful networking between patients and caregivers.

## Abbreviations

CAHPS: Consumer Assessment of Healthcare Providers and Systems
